# Dual-Energy Computed Tomography Analysis of Carotid Calcified Plaques in Stroke Patients

**DOI:** 10.7759/cureus.89645

**Published:** 2025-08-08

**Authors:** Kensaku Yoshida, Reo Ishimoto, Mikio Nakajima, Takuma Higo

**Affiliations:** 1 Neurosurgery, Tokyo Metropolitan Hiroo Hospital, Tokyo, JPN; 2 Neurosurgery, Juntendo University Graduate School of Medicine, Tokyo, JPN; 3 Emergency and Critical Care, Tokyo Metropolitan Hiroo Hospital, Tokyo, JPN

**Keywords:** calcification, cerebral infarction, dual-energy ct, plaque, uremic acid

## Abstract

Background: Vascular calcification represents ectopic deposition of calcium phosphate in the arterial wall. Component analysis of calcifications using dual-energy computed tomography (DECT) has helped to elucidate arteriosclerosis, but reports examining carotid calcified plaque remain lacking. The present study qualitatively evaluated calcifications using DECT in patients with stroke in our institution.

Methods: We performed a single-center, retrospective cohort study based on data obtained from the medical charts of inpatients admitted to Tokyo Metropolitan Hiroo Hospital between March 1 and August 31, 2024. We focused on calcified lesions of carotid plaques on plain CT. Patients were divided into two groups based on the presence or absence of monosodium urate (MSU) in carotid plaques, and in cases where MSU was present, they were further divided into unilateral or bilateral cases. We then analyzed associations between patient background and MSU in carotid plaque.

Results: During the study period, 55 patients were admitted to our facility. Among these, 26 patients with calcified carotid plaque who underwent neck CT were included in this study. Median age was 79 years (interquartile range, 72-85 years) and 17 patients (65.4%) were male. Twenty-three patients (88.7%) were positive for MSU, comprising 10 patients (38.5%) with bilateral lesions and 13 (50%) with unilateral lesions. Patients with hypertension were more likely to show MSU-positive calcified plaque (p = 0.034).

Conclusions: We qualitatively evaluated carotid plaques using DECT in patients with stroke. These findings suggest a potential role of MSU in carotid plaque formation among stroke patients. Future studies should investigate the clinical significance of these findings and potential therapeutic implications.

## Introduction

Vascular calcification refers to pathological deposition of calcium phosphate in the arterial wall [1]. Component analysis of calcifications using dual-energy CT (DECT) has advanced the understanding of arteriosclerosis [2], but studies specifically analyzing carotid calcified plaques remain lacking. This study aimed to qualitatively evaluate calcifications in carotid plaque using DECT among stroke patients at our institution.

While analyzing calcified plaques is crucial, DECT has also gained attention for its ability to detect urate crystals, which has been widely applied in the diagnosis of gout [2]. Although DECT has been utilized to evaluate calcified lesions in atherosclerosis, no studies appear to have assessed its utility with regard to carotid calcified plaques. We report a qualitative assessment of carotid plaque calcification using DECT in stroke patients.

## Materials and methods

Setting and patients

We performed this single-center, retrospective cohort study based on data obtained from the medical charts of inpatients at Tokyo Metropolitan Hiroo Hospital between March 1 and August 31, 2024.

We initially included all patients with cerebrovascular disease admitted to our hospital during the defined study period. Patients who did not undergo CT of the head and neck were then excluded from the study. Patients without calcified lesions in cervical artery plaque as required for analyses were also excluded. All patient diagnoses of cerebrovascular disease were confirmed by echoic examination of the carotid artery.

Data collection

Patient characteristics, medical history, laboratory data on admission, and outcome data were obtained from electronic hospital medical records. Patient characteristics included age, sex, and body mass index. Medical histories included in the present study were hypertension, diabetes mellitus, cardiovascular disease, chronic kidney disease, and admission from a nursing facility. Laboratory data on the day of admission included the following: hemoglobin A1c (HbA1c), serum creatinine, and serum uremic acid.

Imaging with DECT

Neck and chest CT was routinely performed to detect cervical artery stenosis or aortic dissection in our facilities among patients with arteriosclerosis on admission, with the exception of patients who were pregnant or declined CT examination. In addition, DECT of the neck was concurrently performed to exclude deposition of monosodium urate (MSU) in arteriosclerotic cervical calcifications. Detection of MSU deposits was described using the following scores: 0, absent; 1, present unilaterally; 2, present bilaterally. However, quantification of carotid artery MSU deposits was not performed in our cohort. Findings from neck CT were reviewed for the presence of cervical artery calcifications by the radiology department and were evaluated by a neurosurgeon board-certified by the Japan Neurosurgical Society.

Exposure and outcomes

The presence of MSU in the calcified lesion was considered the exposure in the present study. A finding of one or more sites showing MSU in the calcified lesion was interpreted as “present”, while a finding of no MSU was interpreted as “absent”. Patients were thus divided into two groups based on the identification of MSU in carotid plaques, as an Absent group and a Present group. Among the Present group, cases were further subdivided into cases showing unilateral MSU (Unilateral group) or bilateral MSU (Bilateral group). Patient characteristics, risk factors for carotid artery stenosis, MSU deposits and patient background were then compared between groups.

Statistical analysis

We compared patient backgrounds between the Present and Absent groups. Continuous variables are reported as median and interquartile range (IQR) and were compared using the Wilcoxon rank-sum test. Categorical variables are reported as number and percentage and were compared using Fisher’s exact test.

## Results

**Figure 1:**
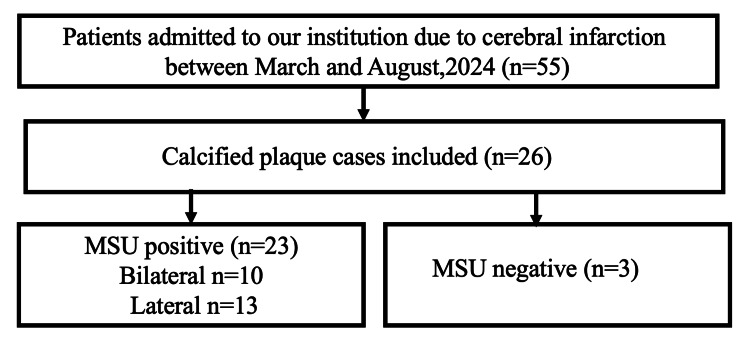
Patient flow chart MSU: monosodium urate

During the study period, 55 consecutive patients were admitted to our facility for cerebral infarction. After applying the inclusion and exclusion criteria, data were extracted from 26 patients (47.2%) (Figure 1).

Clinical background and laboratory findings are provided in Table 1. Median age was 79 years (IQR, 72-85 years) and 17 patients (65%) were male. In terms of the type of cerebral infarction, cardiogenic cerebral embolism was seen in 11 cases, arteriosclerotic thrombosis in 10 cases, and lacunar infarction in five cases.

**Table 1:** Patient characteristics CRP: C-reactive protein, HbA1c: hemoglobin A1c, LDL: low-density lipoprotein, UA: uric acid, BNP: B-type natriuretic peptide, mRS: Modified Rankin scale

	Total (N=26)		Absent (n=3)		Unilateral (n=13)		Bilateral (n=10)		P-value
Age, years	79	(72-85)	84	(72-86)	78	(76-85)	76	(67-82)	0.65
Sex, male	17	(65%)	2	(67%)	7	(54%)	8	(80%)	0.43
Dialysis	2	(8%)	0	(0%)	0	(0%)	2	(20%)	0.18
Hypertension	19	(73%)	0	(0%)	9	(69%)	10	(100%)	0.003
Diabetes	7	(27%)	0	(0%)	5	(38%)	2	(20%)	0.33
Dyslipidemia	9	(35%)	0	(0%)	4	(31%)	5	(50%)	0.26
Hyperuricemia	6	(23%)	0	(0%)	3	(23%)	3	(30%)	0.56
CRP, mg/dl	.15	(.06-.45)	.11	(.04-.45)	.15	(.07-.74)	.15	(.06-.43)	0.84
HbA1c, %	5.95	(5.75-6.65)	6	(5.9-7.2)	6.2	(5.9-6.4)	5.7	(5.4-6.4)	0.24
LDL, mg/dl	95.5	(76-121.5)	88	(59-95)	96	(75-121)	119	(92-143.5)	0.21
UA, mg/dl	6.05	(4.5-6.7)	5.7	(4.2-6.6)	5.6	(4.5-6.7)	6.25	(5.3-7.3)	0.69
Creatinine, mg/dl	.885	(.76-1.07)	.81	(.77-1.05)	.91	(.7-1.06)	.845	(.76-1.46)	0.84
Heart failure	13	(50%)	2	(67%)	7	(54%)	4	(40%)	0.67
BMI, kg/m^2^	22.08	(18.82-23.18)	19.87	(15.5-22.46)	22.49	(19.25-23.39)	22.025	(18.5-22.76)	0.43
BNP, pg/ml	131.3	(48.1-597.2)	211.9	(48.6-597.2)	180	(29.95-481.7)	108.3	(48.1-691.4)	0.97
mRS 3–6	16	(62%)	2	(67%)	9	(69%)	5	(50%)	0.73
mRS 0–2	10	(38%)	1	(33%)	4	(31%)	5	(50%)	

Clinical background characteristics were compared between the Absent and Present groups, the latter of which was further divided into 10 cases in the Bilateral group and 13 cases in the Unilateral group.

No significant differences were found between any groups in age or sex. Among all cases, the positive rate for MSU was 88.5% (23/26 cases). Hypertension was significantly more frequent in the Present group than in the Absent group (p=0.034), but no significant differences were observed for other factors related to atherosclerosis. Likewise, no relationship was identified between uremic acid values and positivity for MSU among patients in this study.

Uric acid levels tended to be higher in the Bilateral group, but this difference was not significant. Mean uric acid level was 5.7 mg/dl in the Absent group, 5.6 mg/dl in the Unilateral group, and 6.25 mg/dl in the Bilateral group. Uric acid level was significantly higher in the Bilateral group. Further, cases treated for hyperuricemia were significantly more frequent in the Present group. Both dialysis patients were in the Present group. There were no cases receiving treatment for hyperuricemia in the Absent group. No significant difference in creatinine levels was noted between the Present and Absent groups.

As a marker of inflammation, C-reactive protein levels were higher in the Present group than the Absent group, but this difference was not significant. HbA1c and low-density lipoprotein (LDL) levels also did not differ significantly between the Present and Absent groups. No differences in heart disease or levels of brain natriuretic protein were observed between the Present and Absent groups. Clinical outcomes also did not differ significantly between the Present and Absent groups.

## Discussion

Many reports have highlighted associations between carotid plaque and hyperlipidemia, emphasizing a need for strict LDL management with various medications, including statins, to control plaque formation [3,4]. However, few reports have discussed the relationship between urate and carotid plaques.

Our research indicated an association between MSU-positive cases and hypertension, but the relationship between hyperuricemia and MSU-positive cases remains unclear.

Two mechanisms of hyperuricemia are hypothesized to explain the impact of urate on blood vessels. The first involves the generation of reactive oxygen species by xanthine oxidase during uric acid production, leading to endothelial dysfunction. In addition, macrophages that phagocytose urate crystals trigger vascular inflammation through the inflammasome pathway [5,6]. The second mechanism involves endothelial damage mediated by urate transporters present in vascular endothelial cells [7,8].

Age may be a factor, as uric acid levels increase with age [9]. Further, uremic acid is presumably involved in responses to vascular damage caused by exposure to hypertension in relation to accumulation of urate. However, some MSU-positive cases do not show hyperuricemia. Held et al. reported that age and positivity for MSU were associated with major cardiovascular events, but not with hyperuricemia [10]. MSU may thus offer a biomarker of cerebrovascular disease.

**Figure 2:**
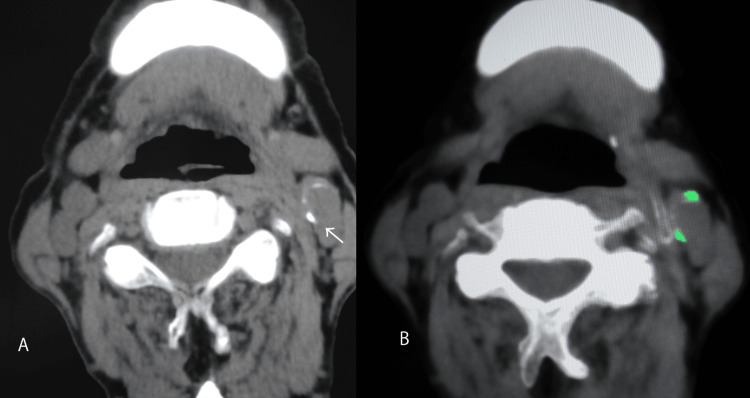
A representative case A 76-year-old man presented suddenly with left middle cerebral artery occlusion with cardiogenic embolism. The white arrow indicated a calcified plaque that was found in the left carotid artery (A). Dual-energy computed tomography (DECT) analysis of the calcified plaque showed that most of them were equivalent to urate indicated by light green (B).

DECT allows more accurate assessment of arterial calcification, particularly fine details of the plaque and degree of calcification, which is difficult with conventional single-energy CT. The analysis of components using DECT has already been clinically applied to the diagnosis of uric acid stones and cardiovascular calcification [11,12]. The present study therefore used DECT to analyze the components of carotid calcified plaques (Figure 2). CT density can vary between abdominal organs and the neck due to differences in the composition of surrounding tissues. However, because the area around carotid plaques consists of soft tissue with low radiopacity, DECT was deemed suitable for diagnosing calcified plaques in this region.

Further improvements in diagnostic accuracy will require analysis of the calcified components of carotid artery plaques in cases requiring carotid endarterectomy.

This represents the first study to establish an association between radiographic evaluations using DECT and carotid plaques in stroke patients. This suggests that uric acid may be involved in the process of plaque formation. Prospective clinical studies are needed to determine whether early therapeutic intervention to address hyperuricemia can help prevent plaque formation.

Several limitations should be noted when interpreting the findings of the present study. First, this investigation was a single-center observational study conducted in Japan. The number of stroke patients in this study was quite limited. Second, the pathology of carotid calcified plaques was not determined in our series. Future work should include analysis of calcified lesions in specimens removed by endarterectomy. Third, unmeasured confounders might have been present between exposures and outcomes, due to the retrospective nature of the study. To resolve this issue, we applied multivariable regression modeling. Using these models, we adjusted numerous variables that were already considered to represent risk or prognostic factors for carotid plaque with uremic acid. Fourth, quantitative assessment of pathological diagnoses was difficult using DECT, so analyses based on quantitative CT of the head will be needed in the future.

## Conclusions

We have reported a qualitative evaluation of carotid plaques using DECT in patients with stroke. Component analysis of carotid calcified plaques in stroke patients using DECT revealed many MSU-positive cases. This finding may suggest the involvement of MSU in the etiology of carotid plaques.
